# Changes in retinal oxygen saturation before and after femtosecond LASIK in adult myopic individuals with anisometropia

**DOI:** 10.1038/s41598-026-37955-4

**Published:** 2026-02-05

**Authors:** Shanshan Ge, Xiaoqi Ma, Xiuli Zhou, Yuehua Zhou

**Affiliations:** 1 Beijing Ming Vision and Ophthalmology, Floors 16-17, Bianyi Fang Building, No. 16, Chongwenmenwai Street, Dongcheng District, Beijing, China; 2 Eye School of Chengdu University of TCM, No. 37 Shierqiao Road, Jinniu District, Chengdu, Sichuan China; 3 International School of Beijing, Anhua Street 10th, Shunyi District, Beijing, China

**Keywords:** Myopic anisometropia, Retinal oxygen saturation, Magnification, Optical, LASIK, Diseases, Health care, Medical research

## Abstract

This study aimed to investigate the relationship between myopia and retinal vascular oxygen saturation, after accounting for optical factors and individual variations. This was a prospective, observational, single-center study. Adults with anisometropia were included. All the participants underwent femtosecond-assisted laser in situ keratomileusis (FS-LASIK), and before and after the surgery, all the patients underwent the examination of visual acuity, intraocular pressure (IOP), spherical equivalent (SE), average keratometry (K), central corneal thickness, and retinal oximeter. A total of 196 adult patients with myopic anisometropia were enrolled. The SE of the less myopic eyes before surgery was − 3.89 ± 3.24 D, which was significantly higher than that of the more myopic eyes − 6.75 ± 3.10 D (*p* < 0.001). The retinal arterial oxygen saturation (SaO_2_) of the less myopic eyes was 94.08 ± 1.61%, which was significantly higher than that of the more myopic eyes 93.36 ± 1.74% (*p* = 0.003). After FS-LASIK, the SaO_2_ of the less myopic eyes (93.29 ± 1.72%) remained significantly higher than that of the more myopic eyes (92.76 ± 1.83%) (*p* = 0.038). In the multivariate regression analysis, the postoperative SaO_2_ was still significantly negatively correlated with axial length (AL) (*B* = -0.252, *P* = 0.009). After excluding optical factors and individual variations, SaO_2_ remains negatively correlated with AL. Myopia exerts an optical magnification effect on SaO_2_.

## Introduction

Retinal vascular oxygen saturation reflects the oxygen saturation of blood in the central retinal artery, vein, and branches. Currently, numerous articles have studied the relationship between myopia and retinal vascular oxygen saturation^1–3^. We have investigated the relationship between myopia and retinal vascular oxygen saturation in individuals with anisometropia, thus ruling out the influence of individual deviations^4^, and conducted a population-based observational cross-sectional study on retinal vascular oxygen saturation in myopic adults^5^. However, the problems of fundus magnification caused by refractive error cannot be imperfectly solved. Our team^6^ have studied the changes in retinal vascular oxygen saturation before and after corneal refractive surgery, confirmed that optical factors have a significant impact on the measurement of retinal arterial oxygen saturation (SaO_2_), and after removing the influence of optical factors, SaO_2_ still showed a significant negative correlation with axial length (AL). In this study, all patients with anisometropia included underwent femtosecond-assisted laser in situ keratomileusis (FS-LASIK) to exclude the influence of optical factors and individual deviations on retinal vascular oxygen saturation measurements, and further investigate the relationship between myopia and retinal vascular oxygen saturation. This was the first study to systematically examine the variations in retinal vascular oxygen saturation in adult patients with myopic anisometropia both prior to and following refractive surgery, offering unprecedented insights into this specific clinical context.

## Methods

Patients who visited Beijing Ming Vision and Ophthalmology from July to December 2023 for corneal refractive surgery were included in this study. All participants signed informed consent forms. The Medical Ethics Committee of the Ineye Hospital of Chengdu University of TCM (2021yh-022) approved this study, and all the procedures adhered to the tenets of the Declaration of Helsinki.

This is a prospective, observational, single-center cohort study. Inclusion criteria: Age: 18 ~ 45 years old, no eye disease and surgery history, no systemic diseases affecting the eyes (such as hypertension, diabetes, autoimmune diseases, etc.), meets the indications for FS-LASIK surgery and agrees to undergo FS-LASIK surgery, SE ≤ -0.50D, CDVA ≤ 0.1(logMAR), the difference in refractive dioptor between the eyes is greater than or equal to 1.50D. Exclusion criteria: Eye surgery and disease history (glaucoma, cataracts, amblyopia, etc.), systemic diseases affecting the eyes, inability to follow up, etc.

All participants underwent preoperative ocular examinations at Beijing Ming Vision and Ophthalmology (one of the teaching bases of Ineye Hospital of Chengdu University of Traditional Chinese Medicine). Preoperative and 1-month postoperative examinations included: visual acuity, intraocular pressure (IOP, CT-800, TOPCON Co., Ltd, Tokyo, Japan), spherical equivalent (SE, subjective refraction after pupil dilatation using Tropicamide Phenylephrine Eye Drops (Mydrin -P; Santen, Osaka, Japan)), average keratometry (K, Topographic Modelling System, TMS-4; Tomey Corporation, Nagoya, Japan) and central corneal thickness (CCT, Haag-Streit Diagnostics, LS 900; Haag-Streit AG, Koeniz, Switzerland), and retinal oximetry examination was performed after mydriasis. Preoperative measurement of AL (Haag-Streit Diagnostics, LS 900; Haag-Streit AG, Koeniz, Switzerland) and fundus examinations after mydriasis were also required.

All surgical procedures were performed by the same experienced surgeon. The Visumax Femtosecond Laser System (Carl Zeiss Meditec AG, Jena, Germany) was used to create corneal flaps with a thickness of 90~110 μm, a diameter of 7.9 mm, and a superior hinge position. After lifting the corneal flap, the excimer laser cutting was conducted using an excimer machine [VISX Star S4 Excimer Laser System (Advanced Medical Optics Inc, Santa Clara, CA, USA)].

Preoperative and one-month postoperative retinal oxygen saturation measurement were performed on participants after dilated pupils. The measuring equipment was a multiwavelength structure–function Coupled Retinal Imager (ROSV-M18; Healthsun Vision, Chengdu, China) and the retinal oxygen saturation analysis equipment was the Healthsun retinal image quantification analysis (HIQA-1, V1). The measurement method of retinal oxygen saturation, the analysis method of the acquired retinal images, and the calculation method of retinal oxygen saturation were all the same as those in the previous articles published by our team^4–6^.

### Statistical analysis

All statistical analyses were performed with IBM SPSS software (Version 25.0; IBM Corp., Armonk, NY, USA). The preoperative general information were described as mean±standard deviation (SD). The independent samples *t*-test was used to compare the vision, SE, CCT, IOP, K, AL, SaO_2_, SvO_2_, and AVD between the eyes before and after corneal refractive surgery, as well as pre- and postoperative. Univariate regression analysis was used to analyze the correlation between SaO_2_ and AL, and multivariate regression analysis was used to analyze the correlation between SaO_2_ and age, IOP, CCT, AL, and K. *P* ≥ 0.05 indicates statistical differences in the results.

Drawing on prior research investigating alterations in retinal oxygen saturation before and after LASIK^6^, the preoperative SaO_2_ was 93.87%, with a mean postoperative value of 93.34% and an estimated standard deviation (SD) of 1.60. significance level of 5% and a power of 90%. The calculated sample size was 157 eyes, corresponding to a cohort of 79 patients with anisometropic myopia. A total of 196 patients with anisometropic myopia were prospectively enrolled in the current study.

## Results

A total of 196 participants with myopic anisometropia who were eligible for FS-LASIK were enrolled in this study. Among these participants, 78 were male and 118 were female. The mean age of the participants was 25.32 ± 6.95 years. The mean SE was − 5.31 ± 3.48D. The mean AL was 25.75 ± 1.40 mm. The mean SaO_2_ was 93.72 ± 1.71%. The mean SvO_2_ was 62.58 ± 4.83%. The mean AVD was 31.14 ± 5.01%. Other preoperative basic data were presented in Table 1.

**Table 1:** General characteristics before the operation.

Characteristic	Mean ± SD	Range
Number (M/F)	196 (78 /118)	
Age (y)	25.32 ± 6.95	(18.00, 44.00)
CDVA (logMAR)	1.10 ± 0.48	(0.00, 2.00)
UCVA (logMAR)	0.01 ± 0.04	(0.00, 0.30)
SE (D)	− 5.31 ± 3.48	(− 12.63, − 0.50)
CCT (µm)	544.02 ± 30.44	(471, 631)
IOP (mm Hg)	16.24 ± 2.78	(10.00, 24.00)
K (D)	43.59 ± 1.31	(40.04, 47.38)
AL (mm)	25.75 ± 1.40	(21.99, 29.61)
SaO_2_ (%)	93.72 ± 1.71	(88.86, 98.22)
SvO_2_ (%)	62.58 ± 4.83	(46.00, 70.00)
AVD (%)	31.14 ± 5.01	(22.20, 47.77)

CDVA, corrected distance visual acuity; UCVA, uncorrected distance visual acuity; SE, spherical equivalent; CCT, central corneal thickness; IOP, intraocular pressure; K, average keratometry; AL, axial length; SaO_2_, retinal arterial oxygen saturation; SvO_2_, retinal venous oxygen saturation; AVD, retinal arteriovenous oxygen saturation difference.

Prior to the surgical procedure, a comparison was made of the basic data of the two eyes in patients with anisometropia. The AL of the less myopic eyes, which was (25.19 ± 1.30) mm, was significantly shorter than that of the more myopic eyes, measuring (26.31 ± 1.27) mm (*P* < 0.001). Moreover, the SaO_2_ of the less myopic eyes, recorded at 94.08 ± 1.61%, was found to be significantly higher than that of the more myopic eyes, standing at 93.36 ± 1.74% (*P* = 0.003). Other pre −  operative data were presented in Table 2.

**Table 2:** Comparisons of the less myopic eyes and the more myopic eyes before operation.

	Less myopic eyes (*n* = 98)	More myopic eyes (*n* = 98)	*P*
CDVA (logMAR)	0.01 ± 0.04	0.01 ± 0.04	0.826
UCVA (logMAR)	0.88 ± 0.52	1.32 ± 0.32	< 0.001*
SE (D)	− 3.89 ± 3.24	− 6.75 ± 3.10	< 0.001*
CCT (µm)	544.23 ± 30.92	543.81 ± 30.11	0.925
IOP (mm Hg)	16.19 ± 2.71	16.29 ± 2.87	0.818
K (D)	43.57 ± 1.34	43.61 ± 1.29	0.841
AL (mm)	25.19 ± 1.30	26.31 ± 1.27	< 0.001*
SaO_2_ (%)	94.08 ± 1.61	93.36 ± 1.74	0.003*
SvO_2_ (%)	63.11 ± 4.74	62.04 ± 4.87	0.121
AVD (%)	30.80 ± 5.04	31.46 ± 4.98	0.356

CDVA, corrected distance visual acuity; UCVA, uncorrected distance visual acuity; SE, spherical equivalent; CCT, central corneal thickness; IOP, intraocular pressure; K, average keratometry; AL, axial length; SaO_2_, retinal arterial oxygen saturation; SvO_2_, retinal venous oxygen saturation; AVD, retinal arteriovenous oxygen saturation difference. *, statistically significant difference.

The comparison of ocular parameters between the two eyes following LASIK was presented in Table 3. Statistically significant differences were observed in IOP, K, and SaO_2_ between the two eyes post-operation (*P* = 0.022, *P* < 0.001, *P* = 0.038). Notably, the SaO_2_ of the less myopic eyes after the procedure (93.29 ± 1.72%) was significantly lower than that of t the more myopic eyes (92.76 ± 1.83%).

**Table 3:** Comparisons of the less myopic eyes and the more myopic eyes after operation.

	Less myopic eyes (*n* = 98)	More myopic eyes (*n* = 98)	*P*
UCVA (logMAR)	− 0.01 ± 0.06	− 0.01 ± 0.08	0.938
SE (D)	0.03 ± 0.60	0.07 ± 0.66	0.650
IOP (mm Hg)	12.49 ± 2.77	11.65 ± 2.28	0.022*
K (D)	39.90 ± 2.40	38.05 ± 2.11	< 0.001*
SaO_2_ (%)	93.29 ± 1.72	92.76 ± 1.83	0.038*
SvO_2_ (%)	62.27 ± 4.57	62.76 ± 4.82	0.465
AVD (%)	31.03 ± 4.80	30.00 ± 4.92	0.143

UCVA, uncorrected distance visual acuity; SE, spherical equivalent; IOP, intraocular pressure; K, average keratometry; SaO_2_, retinal arterial oxygen saturation; SvO_2_, retinal venous oxygen saturation; AVD, retinal arteriovenous oxygen saturation difference; *, statistically significant difference.

The comparison of ocular data before and after the surgery was presented in Table 4. Statistically significant differences were observed in SE, K, and SaO_2_. Specifically, the SaO_2_ decreased significantly compared to its preoperative value (*P* < 0.001).

**Table 4:** Comparation of retinal vascular oxygen saturation before and after operation.

	Preoperative (*n* = 196)	Postoperative (*n* = 196)	*P*
UCDA (logMAR)	1.10 ± 0.48	− 0.01 ± 0.07	< 0.001*
SE (D)	− 5.31 ± 3.48	0.05 ± 0.63	< 0.001*
IOP (mm Hg)	16.24 ± 2.78	12.07 ± 2.57	< 0.001*
K (D)	43.59 ± 1.31	38.97 ± 2.43	< 0.001*
SaO_2_ (%)	93.72 ± 1.71	93.03 ± 1.79	< 0.001*
SvO_2_ (%)	62.58 ± 4.83	62.51 ± 4.69	0.893
AVD (%)	31.14 ± 5.01	30.51 ± 4.88	0.210

UCVA, uncorrected distance visual acuity; SE, spherical equivalent; IOP, intraocular pressure; K, average keratometry; SaO_2_, retinal arterial oxygen saturation; SvO_2_, retinal venous oxygen saturation; AVD, retinal arteriovenous oxygen saturation difference; *, statistically significant difference.

**Fig. 1:**
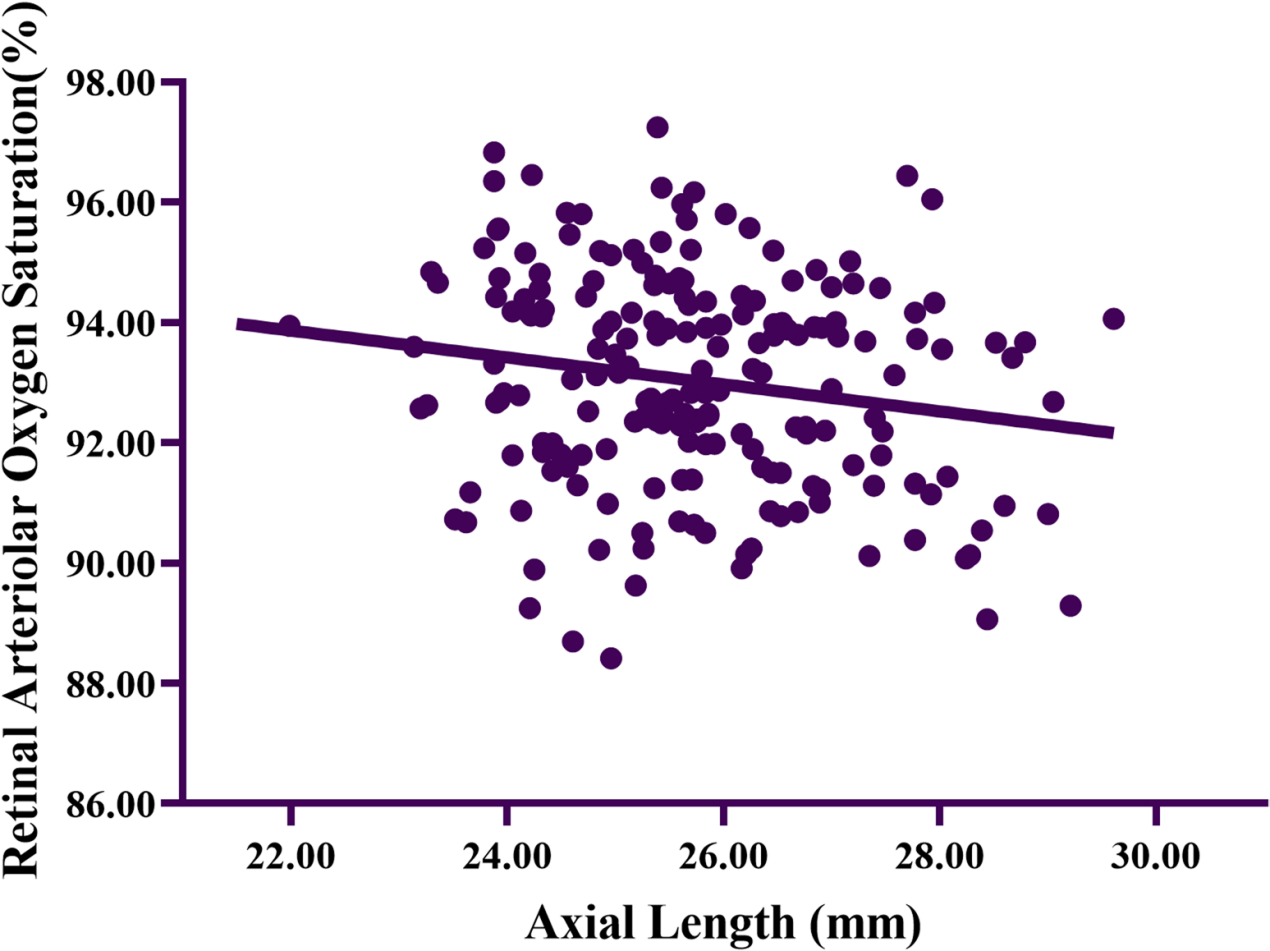
Scatterplot of the relationship between axial length and retinal arterial oxygen saturation. (*r*^*2*^ = 0.031, slope = -0.224, *P* = 0.014)

After LASIK, a pairwise correlation analysis was conducted on variables including age, AL, preoperative IOP, preoperative CCT, and preoperative K. The correlation coefficient between AL and SaO_2_ was − 0.175, with a P - value of 0.014. Table 5 presented the multivariate regression analysis of postoperative SaO_2_. Following LASIK, a significant negative correlation was observed between AL and SaO_2_ (*B* = -0.252, *P* = 0.009). As depicted in Fig. 1, in the univariate regression analysis of the relationship between AL and SaO_2_, postoperative SaO_2_ was significantly negatively correlated with AL (*r²* = 0.031, slope = -0.224, *P* = 0.014).

**Table 5:** Multivariate analysis of the association between Age, AL, IOP, CCT, K and retinal arterial oxygen saturation after FS-LASIK.

Characteristic	Unstandardized coefficients (B)	Standardized coefficients (β)	95% Confidence interval	*R* ^2^	*P*
SaO_2_ (%)				0.057	
Intercept	105.610		(93.094, 118.127)		< 0.001*
Age (y)	0.022	0.087	(− 0.014, 0.059)		0.227
AL (mm)	− 0.252	− 0.198	(− 0.441, − 0.064)		0.009*
CCT (µm)	− 0.066	− 1.326	(− 0.164, 0.033)		0.190
IOP (mmHg)	0.001	− 0.002	(− 0.009, 0.009)		0.981
K (D)	− 0.127	− 1.231	(− 0.330, 0.077)		0.220

SaO_2_, retinal arterial oxygen saturation; AL, axial length; CCT, central corneal thickness; IOP, intraocular pressure; K, average keratometry; *, statistically significant difference.

## Discussion

This study recruited participants with myopic anisometropia. For these myopic patients with anisometropia, LASIK was carried out to eliminate the magnifying effect of refractive errors on the measurement of retinal oxygen saturation, aiming to analyze the relationship between myopia, i.e., AL and retinal oxygen saturation. Our research team had previously investigated the alterations in retinal oxygen saturation before and after LASIK in myopic patients^6^. By enrolling participants with myopic anisometropia who had undergone LASIK in this study, we were able not only to exclude the influence of optical factors on the retinal oxygen saturation measurement results but also to rule out the impact of individual differences on retinal oxygen saturation.

We compared the basic data of the eyes before LASIK. Significant differences were observed in UCVA, SE, AL, and SaO_2_ before the LASIK (Table 2), and these findings were in line with the conclusions of previous research^4^. The outcomes following corneal refractive surgery on both eyes were detailed in Table 3. Post - LASIK, no significant disparities were noted in UCVA and SE between the two eyes. This finding implied that the impact of optical factors on the measurement of retinal oxygen saturation were effectively nullified.

After LASIK surgery, the SaO_2_ in the more myopic eyes group remained significantly lower than that in the less myopic eyes group. In the multivariate regression analysis of postoperative SaO_2_, AL was found to have a significant negative correlation with SaO_2_ (as shown in Table 5 and Fig. 1). This finding confirmed that, in the adult myopic population, independent of the impact of individual deviations and optical factors on measurement, there was an interactive relationship between myopic fundus changes and SaO_2_^4^.

Several prior investigations have also accounted for the impact of optical parameters on the measurement of retinal vascular oxygen saturation^2,7^. In a study^2^ on the relationships between retinal oxygen saturation and refractive error, Lim et al.. attempted to address the issue of optical magnification effect using the Bengtsson formula^8^. A study^7^ investigating changes in retinal vascular oxygen saturation following implantable collamer lens (ICL) surgery observed no significant difference in retinal arterial oxygen saturation levels between the 1-month postoperative and preoperative periods, leading to the conclusion that ICL surgery exerts no impact on retinal oxygen saturation. Notably, however, the study did not address the impact of refractive error on retinal oxygen saturation measurements. Wang et al.^9^reported that laser in situ keratomileusis -induced alterations in retinal microvascular structure showed no significant discrepancy at 1 month postoperatively compared with the preoperative state, with their results analyzed after magnification correction.

In the present study, the retinal oxygen saturation analysis equipment (HIQA-1, V1) did not incorporate correction for refractive error-induced magnification effects prior to retinal vascular oxygen saturation analysis. Consequently, measurements obtained using this oximeter demonstrated a reduction in retinal arterial oxygen saturation following myopia correction surgery, confirming the influence of optical factors on retinal oxygen saturation values and validating the effect of refractive error on the oximeter’s optical magnification.

After excluding optical factors, retinal arterial oxygen saturation still decreased with increasing AL and myopia severity, a phenomenon that was likely attributable to pathophysiological alterations in the myopic retina of adults. AL elongation and progressive myopia induced retinal degenerative changes^10–12^, which reduced the oxygen demand of retinal tissues^13^ and thereby lead to a compensatory decrease in arterial oxygen saturation^14^. Meanwhile, on the other hand, variations in AL led to differences in the optical path length of image formation, which may exert a certain influence on SaO_2_ to some extent^4^. In children with myopia, reduced choroidal blood flow and capillary perfusion^15^ were accompanied by increased retinal vascular oxygen saturation^1,3^, serving as a compensatory mechanism for diminished blood flow. In contrast, adults with myopia exhibited degenerative changes in retinal function: as myopia progressed, oxygen demands decline, resulting in a reduction in retinal arterial oxygen saturation^2,4–6^. A previous study^16^ demonstrated that increased AL was associated with decreased choroidal perfusion, as well as reductions in retinal oxygen delivery and retinal oxygen metabolism. These findings further validated the reduction in oxygen consumption within the myopic retina.

As AL elongated, retinal vessel diameter narrowed^14,17^. Studies have revealed that under both normal physiological conditions and systemic hyperoxia, retinal vessel diameter correlates positively with retinal arterial oxygen saturation^18^. However, with the progression of myopia, the narrowed vessel diameter may result in artificially reduced measurements of retinal oxygen saturation due to factors such as reflected light and artefactual changes^14,19^. Conversely, other studies have suggested a negative correlation between vessel diameter and retinal oxygen saturation. Thus, there may exist reciprocal interactions between retinal vessel diameter and retinal oxygen saturation.

In the present investigation, statistically significant interocular disparities in IOP and K values were identified subsequent to LASIK surgery, primarily attributable to differing degrees of refractive error between the eyes. LASIK corrects myopia by excising corneal stromal tissue to reduce corneal curvature; a higher myopic refractive error necessitates greater stromal ablation, resulting in a more pronounced reduction in corneal curvature. Given the established positive correlation between IOP and CCT^20^, in anisometropic adults who underwent bilateral LASIK, the eye with the higher refractive error exhibited significantly lower postoperative IOP and K values compared to the contralateral eye.

The present study was constrained by the inability to directly obtain specific metrics of choroidal blood flow. Future research focusing on adult myopic populations is therefore merited to more precisely elucidate the potential relationship between choroidal blood flow and retinal vascular oxygen saturation. Furthermore, owing to the absence of direct measurements of vessel diameter changes pre- and post-LASIK, it remains indeterminate whether retinal oxygen saturation is modulated by variations in vessel diameter. Whether LASIK surgery itself exerts an impact on retinal vascular oxygen saturation, and whether long-term changes in refractive status will affect retinal vascular oxygen saturation remain unclear. These constitute critical areas warranting further investigation.

## Conclusions

For the first time, this study successfully eliminated the potential confounding effects of individual deviation and optical magnification on retinal vascular oxygen saturation measurements, while explicitly establishing the correlations between AL, myopia, and SaO_2_. This finding not only advances our understanding of the mechanisms governing the onset and progression of myopia but also offers valuable theoretical underpinnings for the development of more effective myopia prevention and control strategies.

## Data Availability

The datasets used and/or analysed during the current study are available from the corresponding author on reasonable request.

## Abbreviations

AL: Axial length
AVD: Retinal arteriovenous oxygen saturation difference
CCT: Central corneal thickness
CDVA: Corrected distance visual acuity
FS-LASIK: Femtosecond-assisted laser in situ keratomileusis
ICL: Implantable collamer lens
IOP: Intraocular pressure
K: Average keratometry
SaO_2_: Retinal arterial oxygen saturation
SE: Spherical equivalent
SvO_2_: Retinal venous oxygen saturation
UCVA: Uncorrected distance visual acuity
